# Silencing the Myosin Regulatory Light Chain Gene *sqh* Reduces Cold Hardiness in *Ophraella communa* LeSage (Coleoptera: Chrysomelidae)

**DOI:** 10.3390/insects11120844

**Published:** 2020-11-28

**Authors:** Zhenqi Tian, Yan Zhang, Chao Ma, Hongsong Chen, Jianying Guo, Zhongshi Zhou

**Affiliations:** 1 State Key Laboratory for Biology of Plant Diseases and Insect Pests, Institute of Plant Protection, Chinese Academy of Agricultural Sciences, Beijing 100193, China; 82101181184@caas.cn (Z.T.); 82101191195@caas.cn (Y.Z.); 82101171163@caas.cn (C.M.); chenhongsong@gxaas.net (H.C.); guojianying@caas.cn (J.G.); 2 Guangxi Key Laboratory for Biology of Crop Diseases and Insect Pests, Institute of Plant Protection, Guangxi Academy of Agricultural Sciences, Nanning 530007, China

**Keywords:** *MRLC-sqh*, RNA interference, cold hardiness, chill-coma recovery time, *Ophraella communa*

## Abstract

**Simple Summary:**

Cold hardiness is critical to the ability of insects to survive in cold climates and expand their geographical distribution. The molecular mechanisms underlying insect cold tolerance have been well-studied, but many potential genes that may impact these responses, including *MRLC-sqh*, have not been thoroughly evaluated. We first cloned and characterized the *MRLC-sqh* from *Ophraella communa*, an effective biological control agent of *Ambrosia artemisiifolia*, and found that the protein sequence was highly conserved across various Coleoptera insects. The relative expression of *MRLC-sqh* was tissue- and stage-specific, with high levels of expression in the gut and pupal stage of *O. communa*. In addition, the expression of *MRLC-sqh* was shown to decrease after cold shock between 10 and 0 °C and ascend between 0 and −10 °C, but these did not show a positive association between *MRLC-sqh* expression and cold stress. Silencing of *MRLC-sqh* prolonged the chill-coma recovery time in these beetles, suggesting their cold hardiness was reduced in the absence of this protein. Therefore, these results indicate that *MRLC-sqh* may be partly responsible for the regulation of cold-tolerance responses in insects.

**Abstract:**

*Ambrosia artemisiifolia* is a noxious invasive alien weed, that is harmful to the environment and human health. *Ophraella communa* is a biocontrol agent for *A. artemisiifolia,* that was accidentally introduced to the Chinese mainland and has now spread throughout southern China. Recently, we found that upon artificial introduction, *O. communa* can survive in northern China as well. Therefore, it is necessary to study the cold hardiness of *O. communa*. Many genes have been identified to play a role in cold-tolerance regulation in insects, but the function of the gene encoding non-muscle myosin regulatory light chain (*MRLC-sqh*) remains unknown. To evaluate the role played by *MRLC-sqh* in the cold-tolerance response, we cloned and characterized *MRLC-sqh* from *O. communa*. Quantitative real-time PCR revealed that *MRLC-sqh* was expressed at high levels in the gut and pupae of *O. communa*. The expression of *MRLC-sqh* was shown to decrease after cold shock between 10 and 0 °C and ascend between 0 and −10 °C, but these did not show a positive association between *MRLC-sqh* expression and cold stress. Silencing of *MRLC-sqh* using ds*MRLC-sqh* increased the chill-coma recovery time of these beetles, suggesting that cold hardiness was reduced in its absence. These results suggest that the cold hardiness of *O. communa* may be partly regulated by *MRLC-sqh*. Our findings highlight the importance of motor proteins in mediating the cold response in insects.

## 1. Introduction

Temperature is an important abiotic factor that influences the development, survival, and geographical distribution of insects [1,2,3]. In temperate and polar regions, insects are affected by low-temperature-induced stress during winter. Cold injury often causes high mortality, and to counteract this, insects have evolved specific physiological mechanisms to survive in low-temperature conditions. The most relevant physiological response to counteract low temperatures is the accumulation of cryoprotectants, such as sugars, polyols, or amino acids [4]. However, other physiological responses play an important role in surviving in ultra-low temperature conditions [5,6].

Myosin is an actin-dependent motor protein that facilitates muscle contraction, actin cytoskeletal organisation, cell motility, and division in eukaryotes [7,8]. Myosins are found in both muscle and non-muscle cells, with the latter set of proteins referred to as non-muscle myosins. Non-muscle myosin is a motor protein that reversibly binds to actin filaments and generates contractile forces, which are necessary for various phenomena, such as cytokinesis, cell migration, maintenance of cell morphology and polarity, and intracellular vesicle transport [9,10]. All myosins comprise a combination of heavy and light chains, with the light chain components further comprising essential light chains and regulatory light chains [11]. Depending on the source, myosin regulatory light chains (MRLCs) could be classified into MLC-2 (muscle myosin) or MRLC-sqh (non-muscle myosin). MRLC binds Ca^2+^, producing a modulatory effect [12]. The phosphorylation of MRLCs is catalyzed by myosin light chain kinase and is critical to the maintenance of the cytoskeleton, various cellular functions, and muscle contraction in muscle or non-muscle cells [13,14]. Therefore, MRLCs are important for the appropriate physiological functioning of myosin.

Previous studies have demonstrated that MRLCs play several important roles in insect physiology, including flight [15], insecticide resistance [16], and wingbeat frequency [17]. Comparative proteomic and transcriptomic analyses revealed the up-regulation of MRLC protein and mRNA in insects during cold acclimation or rapid cold hardening [18,19,20]. These studies suggest that the MRLC is probably involved in mediating the cold-tolerance response in insects. Recently, a research group found that the differential expression of *MLC-2* corresponds with the reorganization of the muscle fibers in *Drosophila melanogaster* during cold acclimation [18], but whether *MRLC-sqh* plays a role in insect cold hardiness remains unclear. Therefore, it is necessary to explore the function of *MRLC-sqh* in insect cold hardiness.

Common ragweed, *Ambrosia artemisiifolia*, is a noxious invasive alien species that is harmful to the environment as well as to human health [21,22]. Its abundance threatens crop yields and biodiversity, and the pollen produced by *A. artemisiifolia* is a key cause of allergic rhinitis and asthma [22,23]. Unsurprisingly, given the growing effects of climate change, its geographical distribution is continuously expanding, thereby necessitating the careful development of management programs [24,25]. *Ophraella communa* is an effective biocontrol agent for *A. artemisiifolia* [26] and has been successfully used to reduce *A. artemisiifolia*-associated negative effects on human health and economy [27]. *O. communa* is native to North America and was accidentally introduced to the Chinese mainland in 2001 [28]. Now, it has spread throughout the southern parts of China. It was introduced to Beijing (39.98° N, 115.97° E) from Laibin (23.62° N, 109.37° E) in 2012 [29]. This suggests that *O. communa* can survive the colder winters of northern China, making it a viable intervention for ragweed. Therefore, it is important to understand the cold-tolerance response of *O. communa*.

Previous studies have demonstrated a link between the cold hardiness of *O. communa* and the activity of protective enzymes and accumulation of cryoprotectants [30,31,32]. However, the role played by *MRLC-sqh* in mediating this phenomenon remains unknown. Here, we tried to evaluate the function of the gene coding for the myosin regulatory light chain sqh (MRLC-sqh) with respect to the cold-tolerance response of *O. communa*. First, we cloned and characterized *MRLC-sqh* and evaluated its expression in different tissues and at various developmental stages. Then, we determined the expression of the *MRLC-sqh* in response to different temperatures, and finally, we silenced *MRLC-sqh* using RNA interference (RNAi) and evaluated the chill-coma recovery time (CCRT) of the treated beetles. The results of these assays reveal a novel function of *MRLC-sqh* in the *O. communa* cold-hardiness response that may be used as a reference when exploring new aspects of cold tolerance. Our findings highlight the importance of motor proteins in mediating the cold response in insects.

## 2. Materials and Methods

### 2.1. Insect Source

Both female and male adult *O. communa* were collected from *A. artemisiifolia* plants growing in Laibin City, Guangxi Zhuang Autonomous Region, China (23.62° N, 109.37° E) in July 2019. They were reared on *A. artemisiifolia* plants in cages in a laboratory at the Langfang Experimental Station, Chinese Academy of Agricultural Sciences, Langfang City, Hebei Provence, China (39° N, 116° E). The following rearing conditions were used: temperature, 26 ± 1 °C; relative humidity, 70 ± 5%; photoperiod, 14:10 light/dark cycle. These conditions are suitable for the growth and development of *O. communa* [2].

### 2.2. RNA Extraction, cDNA Synthesis, and Gene Cloning

Total RNA was extracted from 3-day-old adult *O. communa* samples using TRIzol (Invitrogen, Carlsbad, CA, USA) according to the manufacturer’s instructions. First strand cDNA synthesis was performed using 1.0 µg total RNA and the One-step Reagent Kit with gDNA Eraser (TransGen Biotech, Beijing, China). Subsequently, according to the sequence obtained from the previous transcriptome data (shown in the Supplementary Materials), a pair of specific primers were designed using Primer Premier 5 (PREMIER Biosoft International, Palo Alto, CA, USA; Table 1). They were used to amplify the *MRLC-sqh* sequence using the cDNA as a template. The amplification product was then purified using the Monarch Gel Extraction kit (NEB, Ipswitch, MA, USA) and cloned into a Trans1-T1 clone vector (TransGen Biotech) and sequenced [33].

### 2.3. Sequence Analysis

Multiple sequence alignment of *MRLC-sqh* and its four orthologues from Coleoptera insects was performed using DNAMAN v7.0 (Lynnon Corporation, San Ramon, CA, USA). A protein-protein Basic Local Alignment Search Tool (BLASTP) search was performed using the translated potential MRLC-sqh protein sequences from *O. communa* as queries in NCBI. The amino acid sequences of the four orthologues were downloaded from NCBI. The physical and chemical properties of *O. communa* MRLC-sqh were predicted based on the amino acid sequence using ExPASy (http://web.expasy.org/protparam/) and the conserved functional domains were predicted using the InterProScan database (http://www.ebi.ac.uk/interpro/search/sequence/). ClustalW was used to perform multiple sequence alignment of the predicted MRLC-sqh sequence with that of MRLC-sqh from 49 insect species (retrieved from NCBI). A phylogenetic tree was constructed using the neighbor-joining method with 1000 bootstrap replicates in MEGA v7.0 (https://www.megasoftware.net/).

### 2.4. Quantitative Real-Time PCR (qPCR) Analysis

Seven tissues were dissected from the mature (3-day-old) adult insects; these included the head, thorax, Malpighian tubule (MT), fat body, ovary, gut, and spermaries (SP). Further, seven developmental stages were also sampled and frozen in liquid nitrogen; these included the egg, 1st instar larvae (L-1), 2nd instar larvae (L-2), 3rd instar larvae (L-3), pupae, amd 3-day-old adult females (Female) and males (Male). Total RNA was extracted from these tissues and developmental stages and cDNA was synthesized as described above. The primers for the qPCR assays were designed using Beacon Designer 8; these have been summarized in Table 1. *Ribosomal protein L4* (*RPL4*) was used as the reference gene [34]. The expression profiles were analyzed using the ABI 7500 system (Applied Biosystems, Foster City, CA, USA) and a qPCR SYBR Green Master Mix (Yeasen, Shanghai, China). The PCR conditions were as follows: 95 °C for 5 min, 40 cycles of 95 °C for 10 s, and finally, 60 °C for 30 s, followed by a melting curve analysis. The melting curves were checked to test the purity of qPCR reaction. The efficiency of the primers was also validated before gene expression analysis. Each treatment contained three technical replicates and three biological replicates.

### 2.5. Low-Temperature Stress Treatment

To evaluate the response of *MRLC-sqh* during conditions of low-temperature stress, we selected three 3-day-old adult females and placed them in 1.5-mL centrifuge tubes and exposed them to the following conditions: 10, 5, 0, −5, or −10 °C for 3 h in a cold water bath (Huber, Berching, Germany). For control treatment, the centrifuge tube was placed in a growth chamber at a temperature of 25 °C for 3 h. After treatment, the insects were frozen in liquid nitrogen and used for qPCR. The extraction of total RNA from different treatments, cDNA synthesis, and qPCR analysis were conducted as described above.

### 2.6. RNAi

Double-stranded RNA (dsRNA) mimics were designed using Primer Premier 5.0 (Table 1) and synthesized using the MEGAscript T7 High Yield Transcription Kit (Ambion, Austin, TX, USA). To indicate that the injection and the response of immune system would not affect the expression of target gene and the results in study, we designed the primers of GFP-specific dsRNA (ds*GFP*) [33,35]. The newly emerging female adults were injected at the pronotum with 1 µg ds*MRLC-sqh* using a PLI-100 Pico-Injector (Harvard Apparatus, Holliston, MA, USA), which we manipulated with an MP-255 Micromanipulator (Sutter, Novato, CA, USA) under a stereomicroscope. Simultaneously, we set control groups, including those injected with ds*GFP* and uninjected treatment. Due to the high interference efficiency 48 h after dsRNA injection [33], so forty-eight hours after dsRNA injection, 10 beetles were injected with either ds*MRLC-sqh* (5) or ds*GFP* (5) and frozen in liquid nitrogen and stored at −80 °C until analysis of *MRLC-sqh* mRNA expression.

### 2.7. Measurement of CCRT

Cold hardiness in these beetles was evaluated by measuring the recovery time following a chill coma induced by cold shock [35,36,37]. These assays also used three treatments, which were either injected with ds*MRLC-sqh*, injected with ds*GFP*, or uninjected. These individuals were placed in a centrifuge tube and exposed to a temperature of −10 °C for 10 min to induce cold shock. After the cold shock, the insects were placed on a piece of paper and allowed to recover at room temperature (25 °C). CCRT was recorded as the time from placement on the paper to the recovery of the ability of the beetle to spontaneously regain an upright position [36]. Each treatment was performed using three replicates of 18–20 beetles each.

### 2.8. Statistical Analyses

CT values were the average of the three technical replicates and three biological replicates. Relative mRNA expression was calculated using the 2^−ΔΔCT^ method [ΔΔCT = (Ct_target_ − Ct_reference_)_treatment_ − (Ct_target_ − Ct_reference_)_control_] [38,39] and the data were analyzed using a one-way analysis of variance (ANOVA) and an LSD test with SAS 8.1 (SAS Institute, Cary, NC, USA). Data are represented as the mean ± SE. A *p*-value of <0.05 was considered significant.

## 3. Results

### 3.1. Cloning and Sequence Analysis of MRLC-sqh

**Figure 1:**
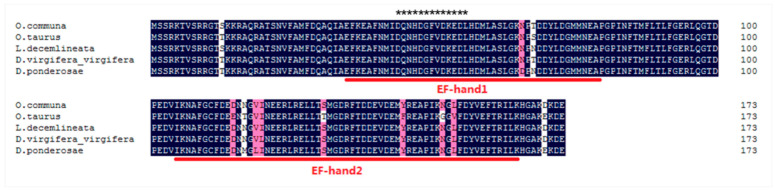
Multiple sequence alignment of myosin regulatory light chain sqh (MRLC-sqh) from *O. communa* and four other Coleoptera insects. The conserved domains are underlined with red lines and the Ca^2+^-binding sites within the first EF-hand are marked with an “*”. *O. communa*, *Ophraella communa*; *O. taurus*, *Onthophagus taurus*; *L. decemlineata*, *Leptinotarsa decemlineata*; *D. virgifera*, *Diabrotica virgifera*; *D. ponderosae*, *Dendroctonus ponderosae*.

**Figure 2:**
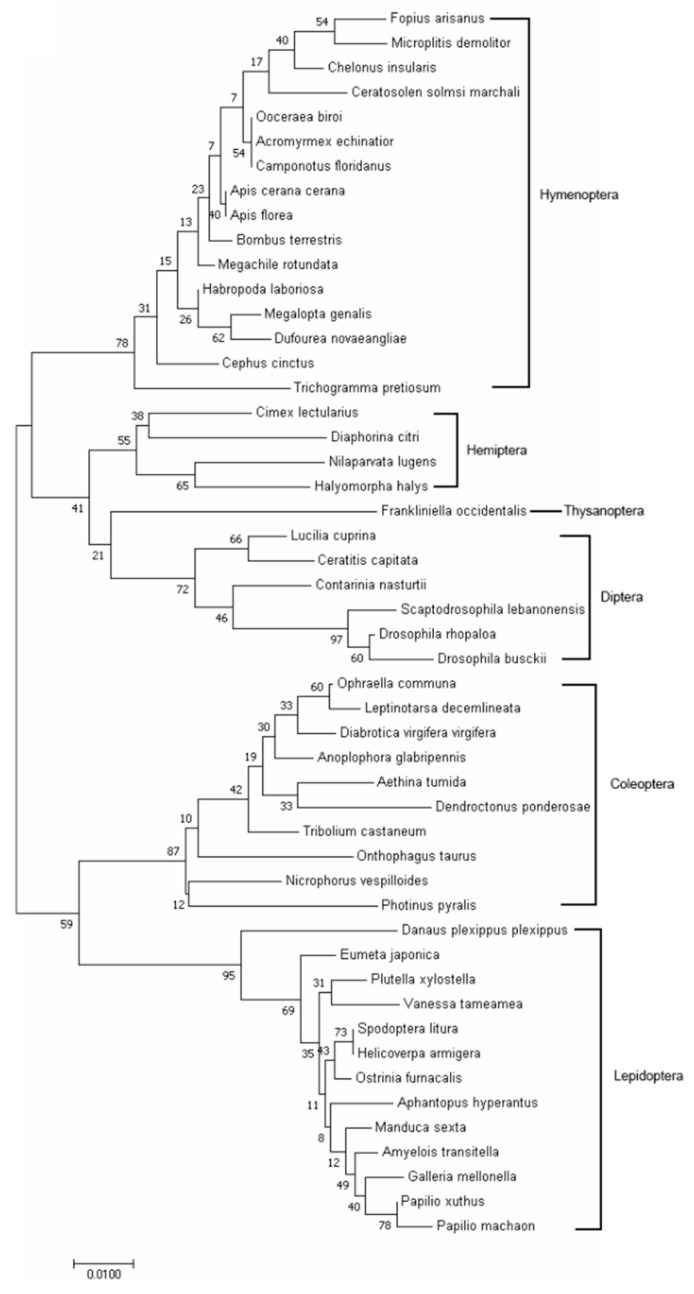
Phylogenetic tree depicting the relationships between the MRLC-sqh proteins of various insects, constructed using the neighbor-joining method with 1000 bootstrap replications.

The full-length cDNA sequence of *MRLC-sqh* from *O. communa* was successfully cloned (GenBank accession number: MT978153). The complete open reading frame was found to comprise 525 bp, encoding 174 amino acids (Figure 1), with a predicted isoelectric point (pI) of 4.63 and a molecular mass of 19.92 kDa. The protein was predicted to include two conserved EF-hand domains, with the first EF-hand frame (Figure 1) containing several Ca^2+^-binding sites (DQNHDGFVDKEDL). The multiple sequence alignments indicate that MRLC-sqh is highly conserved in insects (Figure 1) and the predicted *O. communa* MRLC-sqh shared 94.83%, 99.43%, 98.85%, and 96.55% sequence identity with the MRLC-sqh of *O. taurus*, *Leptinotarsa decemlineata*, *Diabrotica virgifera*, and *Dendroctonus ponderosae*, respectively. Phylogenetic analyses showed that MRLC-sqh proteins of insects from the same order cluster into the same branch (Figure 2).

### 3.2. Expression Profile of MRLC-sqh in Various Tissues and Developmental Stages

**Figure 3:**
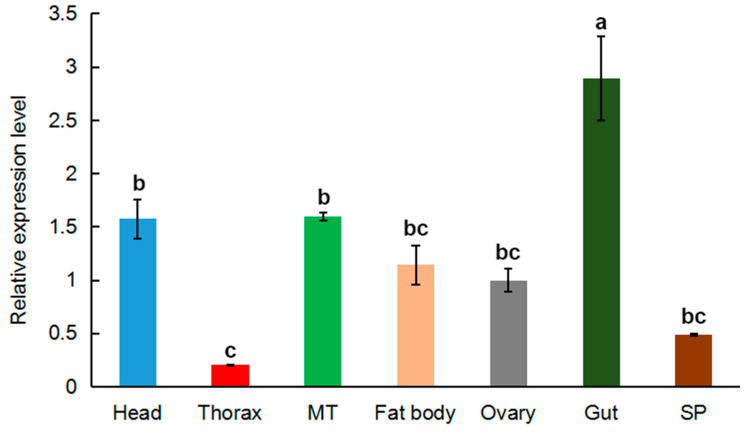
The relative expression of *MRLC-sqh* in the tissues of adult *O. communa*. The expression fold changes were related to the ovary. The transcript level of *MRLC-sqh* was normalized against that of r*ibosomal protein L4* (*RPL4)*. Relative expression is represented as mean ± SE. Abbreviations: MT, Malpighian tubule; SP, spermaries. Bars with different lowercase letters are significantly different at *p* < 0.05.

The relative expression of *MRLC-sqh* in tissues from adult (3-day-old) *O. communa* was determined using qPCR. *MRLC-sqh* mRNA was expressed in all the tissues evaluated, with the highest expression in the gut and lowest expression in the spermaries (SP) and thorax (Figure 3).

**Figure 4:**
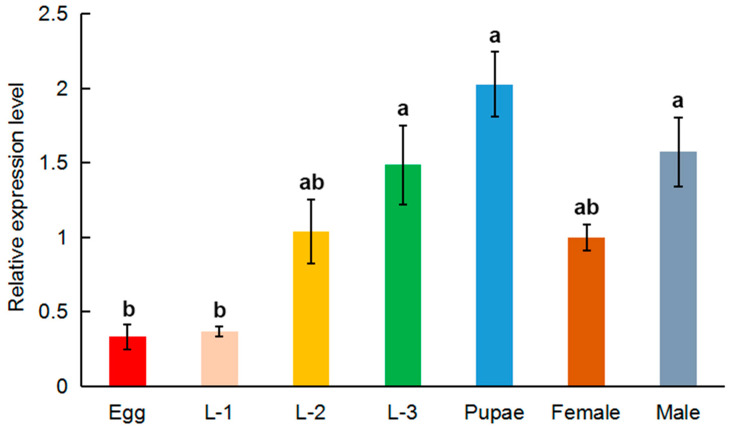
The expression profile of *MRLC-sqh* during various stages of *O. communa* development. The expression fold changes were related to the female. The transcript level of *MRLC-sqh* was normalized against that of *RPL4*. Relative expression is represented as mean ± SE. L-1: 1^st^-instar larvae; L-2: 2^nd^-instar larvae; L-3: 3^rd^-instar larvae; Female: 3-day-old female adults; Male: 3-day-old male adults. Bars with different lowercase letters are significantly different at *p* < 0.05.

The expression patterns of *MRLC-sqh* at the various developmental stages of *O. communa* were also evaluated (Figure 4). *MRLC-sqh* was widely expressed in all developmental stages, including the egg, larvae, pupae, and adults (both male and female). The relative expression of *MRLC-sqh* started increasing from the 1st-instar-larvae stage and reached its peak at the pupal stage.

### 3.3. MRLC-sqh Expression in Response to Exposure to Low Temperature Conditions

**Figure 5:**
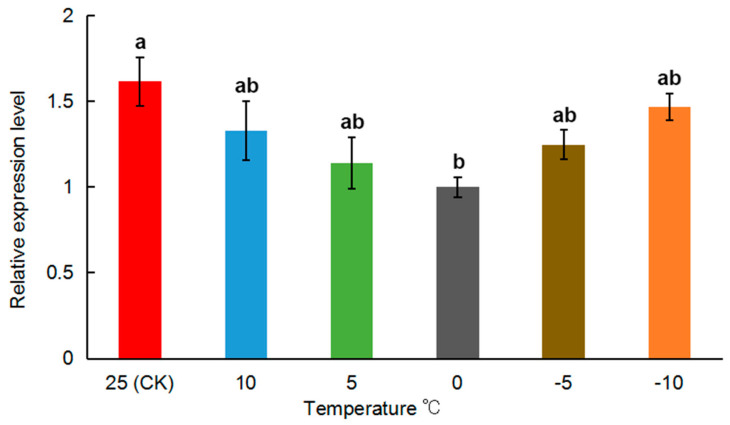
Relative expression of *MRLC-sqh* in *O. communa* after exposure to different temperature conditions over a 3-h period. The expression fold changes were related to the 0 °C. The transcript level of *MRLC-sqh* was normalized against that of *RPL4*. Relative expression is represented as mean ± SE. The values marked with different letters are significantly different based on one-way ANOVA and LSD analyses.

The relative expression of *MRLC-sqh* in response to cold shock was determined by qPCR (Figure 5). *MRLC-sqh* mRNA showed a gradual decrease in expression from 25 (CK) to 0 °C, and an increase from 0 to −10 °C. The relative expression of *MRLC-sqh* at 0 °C was significantly lower than that at 25 °C (*p* < 0.05), but no significant difference was observed in its expression between 0 and −10 °C (*p* > 0.05).

### 3.4. Effect of RNAi on MRLC-sqh Expression and CCRT

**Figure 6:**
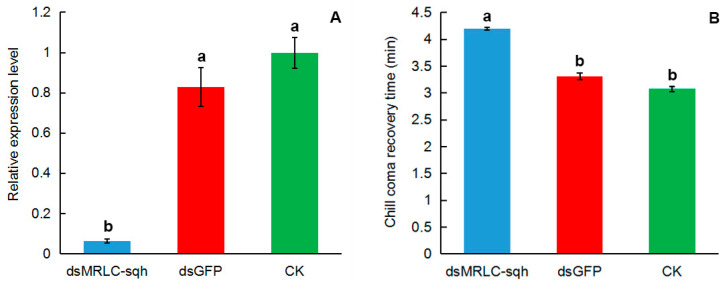
Relative expression levels of *MRLC-sqh* (**A**) and chill-coma recovery time (CCRT) (**B**) in *O. communa* after double-stranded RNA (dsRNA) treatment. CK represents the untreated controls. The expression fold changes were related to the CK. The transcript level of *MRLC-sqh* was normalized against that of *RPL4*. Relative expression is represented as mean ± SE. The bars marked with different lowercase letters are significantly different based on one-way ANOVA and LSD analyses.

*MRLC-sqh* expression was evaluated 48 h after dsRNA treatment using qPCR. The relative expression of *MRLC-sqh* reduced by nearly 90% following the injection with ds*MRLC-sqh* compared to that observed in the ds*GFP*-injected and CK (untreated) controls (Figure 6A). The CCRT was significantly longer in the ds*MRLC-sqh*-treated adults than in either of the controls (4.2 vs. 3.3 vs. 3.08 min, *p* < 0.05; Figure 6B).

## 4. Discussion

Cold hardiness is an important factor that allows insects to survive over winter and expand their geographical range. Many mechanisms are involved in the cold hardiness of insects [6], and previous studies have shown that many genes can regulate cold tolerance in insects. However, the role of *MRLC-sqh* in insect cold tolerance has not been investigated. Non-muscle myosin powers the actomyosin cytoskeleton and performs crucial functions in various cellular processes [9,10]. Myosin activation is regulated by the phosphorylation of MRLC, and this makes this protein a critical muscle tissue component. Recent studies have reported the up-regulation of MRLC in insects during cold acclimation and rapid cold hardening [18,19,20]. These findings indicate that MRLC may be, in some way, responsible for the cold hardiness of insects. Our results provide experimental evidence that *MRLC-sqh* might regulate cold hardiness in insects.

In this study, we reported the full cDNA sequence of *MRLC-sqh* in *O. communa*. Two EF-hand domains were detected in the MRLC-sqh protein, and a single Ca^2+^-binding region was identified in the first EF-hand frame. Our results are consistent with those of prior studies in MLC-2 [16,40,41,42], and the predicted MRLC-sqh shared a 98.05% sequence identity with MRLC-sqh from other insects. This suggests that the function of MRLC-sqh is relatively conserved across species. Phylogenetic analysis revealed that MRLC proteins are highly conserved within the same order as well as across insect orders. These results suggest that MRLC-sqh has a highly conserved structure and function over different evolutionary time scales.

*MRLC-sqh* was widely expressed in all the tissues and developmental stages evaluated in this study (Figure 3 and Figure 4). The relative expression of *MRLC-sqh* was the lowest in the thorax; this differed from *MLC-2*. A high expression of *MLC-2* was validated in the thorax, likely due to the higher proportion of muscles in this tissue [41]. Interestingly, *MRLC-sqh* was expressed at high levels in the gut. Based on the fact that *MRLC-sqh* expression has been detected in the epithelial cells of many other tissues, we hypothesize that *MRLC-sqh* might be primarily expressed in the epithelial cells of the midgut [43], but this needs to be confirmed by further experiments. In addition, the expression of *MRLC-sqh* was marginally higher in the ovaries compared to that in the spermaries, but this difference was not significant. This pattern is similar to that observed for *MLC-2* expression in *A. pernyi* [40]. This is probably because ovarian development is an active process. The expression of *MRLC-sqh* gradually increased between the 1^st^- and 3^rd^-instar larval stages, and the expression was highest in the pupal stage, likely linked to the critical function of non-muscle myosin during the morphogenesis of multicellular organisms [43]. This result is consistent with the fact that the function of non-muscle myosin involves cell motility and division [9,10]. The larval and pupal stages are important periods of growth and development and involve cell migration and division. This is especially true for pupae, which undergo drastic remodeling to produce the new tissues and organs of the mature adults.

The expression profile of *MRLC-sqh* was evaluated in response to low-temperature-induced stress. *MRLC-sqh* expression significantly declined as the temperature dropped from 25 to 0 °C, but it ascended from 0 to −10 °C. (Figure 5). There was no significant difference between 0 and −10 °C; even an obvious trend was observed in the data. These findings demonstrate that *MRLC-sqh* expression does not simply decline or ascend with the decrease in temperature. The differential expression of *MRLC-sqh* was similar to that recorded for actin and microtubules in insects and plants during cold stress. Actin or microtubules usually undergo disassembly and reassembly to maintain their cellular structure and avoid depolymerization at low temperatures [44,45], and this process is driven by the actin–myosin motor [46]. Furthermore, the actin network can also determine the activity of myosin [47], suggesting that this relationship may be regulated by *MRLC-sqh* at low temperatures. Therefore, it can be speculated that the decrease in *MRLC-sqh* expression from 25 to 0 °C is related to the disassembly of actin, and correspondingly, the increase from 0 to −10 °C is related to the reassembly of actin, due to the combined effects of disassembly and reassembly. There was no significant difference in *MRLC-sqh* expression between 25 and −10 °C, indicating that most actins were reassembled, but still, a little was disassembled.

We can see that the CCRT of *O. communa* injected with ds*GFP* is slightly higher than that of the CK (non-injected treatment), and there is no significant difference between them (Figure 6B). As such, the side effects of injection or immune system reactions on CCRT were slight. When *MRLC-sqh* was silenced using RNAi, the CCRT of the *O. communa* was significantly delayed (Figure 6B), indicating that the cold hardiness of *O. communa* had significantly decreased. CCRT is a widely used parameter to estimate the cold hardiness of insects [36,37,48,49,50]; it depends on the reestablishment of ion homeostasis and normal membrane potential [5]. Therefore, CCRT is a critical measure of cellular integrity and homeostasis. Induction of cell death during cold injury indicates damage to the cellular membranes, a phenomenon that may be caused by the dissociation or denaturation of the cytoskeleton [51]. Myosin is involved in cytoskeletal regulation and protects cells under cold stress [52]. One study even suggested that non-muscle myosin aids cell membrane repair by coordinating the movement of intracellular vesicles to the membrane injury sites, thereby facilitating patch creation [53]. As MRLC-sqh is critical to the physiological activity of non-muscle myosin, it makes sense that its inhibition would limit these remodeling responses. When *MRLC-sqh* was silenced, non-muscle myosin lost its ability to bind Ca^2+^ and was unable to undergo phosphorylation, an event that affected its ability to bind to the N-terminus of the light chain and facilitate actin binding [12]. As a result, cellular contraction and associated phenomena, such as cytokinesis, could not progress, thereby reducing the organizational flexibility of the actin cytoskeleton [14] and reducing cold hardiness. Taken together, these results support the hypothesis that *MRLC-sqh* is critical for the regulation of cold hardiness in insects.

We found a negative association between *MRLC-sqh* expression and low temperatures, from 0 to −10 °C (Figure 5); therefore, we suggest that the potential mechanism for the regulation of cold hardiness in *O. communa* does not involve low-temperature-mediated up-regulation of the gene. Given the various functions of non-muscle myosin in cellular processes, and the binding of *MRLC-sqh* to Ca^2+^, we believe that *MRLC-sqh* is one of the genes whose regular function is needed for proper chill-coma recovery. It can regulate other resistances of insects, besides cold resistance, because of the mechanistic overlap between these stresses. The *capability* (*capa*) neuropeptide gene, for example, is a desiccation- and cold stress-responsive gene [54]. The functions of the *MRLC-sqh* response to other stresses should be detected by further studies.

## 5. Conclusions

To our knowledge, this study describes the full-length cDNA sequence and characteristics of *O. communa MRLC-sqh* for the first time. We found that *MRLC-sqh* was highly expressed in the gut and at the pupal stage. Importantly, we revealed that when the *MRLC-sqh* gene was silenced by RNAi, the CCRT of *O. communa* was prolonged, which suggests that *MRLC-sqh* could regulate the cold hardiness. However, we did not observe a positive association between *MRLC-sqh* expression and cold stress under the conditions of 0 °C above and 0 °C below. This indicated that the potential mechanism for *MRLC-sqh* to regulate the cold hardiness of *O. communa* does not involve lower-temperature-mediated up-regulation of the gene. Further research can focus on this potential mechanism. Our results may provide new insights for future studies on the insect cold-tolerance response.

## Figures and Tables

**Table 1:** Primers used in this study.

Primer Name	Primer Sequence (5′ to 3′)
**RT-PCR**	
*MRLC-sqh*-F	ATGTCTTCCCGTAAAACT
*MRLC-sqh*-R	TTACTGCTCATCTTTATCTT
**qPCR**	
*MRLC-sqh*-F	CGTCTTCAAGGTACTGATCC
*MRLC-sqh*-R	AATGGGAGCCTCTCTGTA
*RPL4*-F	TGTGGTAATGCTGTGGTAT
*RPL4*-R	TCTAGCACTGCATGAACA
**dsRNA**	
ds*MRLC-sqh*-F	TAATACGACTCACTATAGGGAACTGTAAGTCGTCGTG
ds*MRLC-sqh*-R	TAATACGACTCACTATAGGGCATCAGTGAATCTATCTCCC
ds*GFP*-F	TAATACGACTCACTATAGGGTGAGCAAGGGCGAGGAG
ds*GFP*-R	TAATACGACTCACTATAGGGCGGCGGTCACGAACTCCAG

## Supplementary Materials

The following are available online at https://www.mdpi.com/2075-4450/11/12/844/s1, Table S1: Transcriptome data of *MRLC-sqh* for design of specific primers.
