# Supplementary material for: Silencing the Myosin Regulatory Light Chain Gene sqh Reduces Cold Hardiness in Ophraella communa LeSage (Coleoptera: Chrysomelidae)
Source: Insects. 2020 Nov 28;11(12):844. doi: 10.3390/insects11120844 (PMC7768443; doi:10.3390/insects11120844)
Supplement: Supplementary file 1 [file insects-11-00844-s001.doc]

Table S1: Transcriptome data of MRLC-sqh for design of specific primers

>c81217_g1;orf1 len=597 frame:-1 start:1022 end:426 gi|91079212|ref|XP_966680.1| PREDICTED: myosin regulatory light chain sqh [Tribolium castaneum]>gi|642916704|ref|XP_008192258.1| PREDICTED: myosin regulatory light chain sqh [Tribolium castaneum]>gi|270004989|gb|EFA01437.1| hypothetical protein TcasGA2_TC030667 [Tribolium castaneum]

TGCTCTATATTTAATTTAATTAATAATTTTAGTTATTGTTTGATTTTAGCGGATAACATATTTAAAAGCATAATGTCTTCCCGTAAAACTGTAAGTCGTCGTGGAACTAGTAAAAAACGTGCTCAAAGAGCAACATCCAATGTATTTGCTATGTTTGATCAGGCACAAATTGCAGAATTCAAAGAGGCATTTAATATGATTGACCAAAACCATGATGGCTTTGTTGACAAAGAAGATTTACATGACATGTTGGCTTCTTTAGGTAAAAACCCTACTGATGATTATTTAGATGGCATGATGAATGAAGCACCTGGTCCAATAAACTTTACTATGTTTTTAACACTCTTTGGTGAACGTCTTCAAGGTACTGATCCAGAAGATGTCATAAAAAATGCTTTTGGTTGTTTTGATGAGGACAATAATGGAGTAATTAATGAAGAACGACTTCGAGAACTCTTAACATCAATGGGAGATAGATTCACTGATGATGAAGTTGATGAGATGTACAGAGAGGCTCCCATTAAGAATGGATTGTTTGATTATGTTGAATTTACACGTATTTTGAAACATGGTGCTAAAGATAAAGATGAGCAGTAA
